# Serum high mobility group box 1 protein levels are not associated with either histological severity or treatment response in children and adults with nonalcoholic fatty liver disease

**DOI:** 10.1371/journal.pone.0185813

**Published:** 2017-11-02

**Authors:** Katherine P. Yates, Ross Deppe, Megan Comerford, Howard Masuoka, Oscar W. Cummings, James Tonascia, Naga Chalasani, Raj Vuppalanchi, for the NASH CRN

**Affiliations:** 1 Bloomberg School of Public Health, The Johns Hopkins University, Baltimore, Maryland, United States of America; 2 Department of Medicine, Indiana University School of Medicine, Indianapolis, Indiana, United States of America; INRA, FRANCE

## Abstract

**Aim:**

Serum high mobility group box 1 protein (HMGB1) is a proinflammatory molecule that could potentially serve as a biomarker for non-alcoholic fatty liver disease (NAFLD) and non-alcoholic steatohepatitis (NASH) due to its correlation with degree of liver fibrosis. The aim of the current study was to examine the cross-sectional and longitudinal relationships between serum HMGB1 levels and liver histology in adults and children with NAFLD participating in two large randomized controlled trials.

**Methods:**

Serum HMGB1 levels were measured at various time points in adults and children with NAFLD, who participated in PIVENS and TONIC clinical trials respectively. PIVENS trial compared vitamin E or pioglitazone to placebo in adults whereas TONIC trial compared vitamin E or metformin to placebo in children. Participants had liver biopsies at baseline and the end of treatment (96 weeks), and liver histology was reviewed by a central committee of study pathologists.

**Results:**

In the cross-sectional analyses (n = 205 for PIVENS and 109 for TONIC), there was no significant relationship between serum HMGB1 levels and histological features such as steatosis, ballooning, inflammation, fibrosis, or presence of steatohepatitis in either adults or children. Serum HMGB1 levels did not change significantly during treatment either with placebo, vitamin E therapy (*P* = 0.81) or pioglitazone (*P* = 0.09) in the PIVENS trial. Similarly, serum HMGB1 levels did not change significantly during treatment either with placebo, metformin (*P* = 0.15) or vitamin E (*P* = 0.23) in the TONIC trial. In the longitudinal analyses (n = 105 for PIVENS and 109 for TONIC), changes in serum HMGB1 levels did not correlate with histologic improvement or resolution of NASH in either adults or children. There was no relationship between serum HMGB1 and ALT levels in either adults or children with NAFLD.

**Conclusion:**

Serum HMGB1 levels were not associated with histological severity or treatment response in either children or adults with NAFLD.

## Introduction

Non-alcoholic fatty liver disease (NAFLD) is a common liver condition and is estimated to affect one in three adults in the United States.[1] Its prevalence in children is also increasing in parallel with childhood obesity.[2–4] The severity of NAFLD is varied and could range from simple hepatic steatosis or non-alcoholic fatty liver (NAFL) to nonalcoholic steatohepatitis (NASH), a progressive condition that could lead to cirrhosis, hepatocellular cancer or liver failure.[5] Since NASH is a histologic diagnosis, a definitive diagnosis requires the patient to undergo a liver biopsy.[5] Currently, the therapeutic end point in clinical trials for NASH requires a liver biopsy at enrollment and after completion of the study in order to show resolution of NASH with therapeutic intervention.[6] A non-invasive biomarker for the diagnosis and severity of NAFLD that can also change dynamically with histological improvement in a treatment trial is very desirable and currently an unmet need.

In addition to metabolic factors, sterile inflammation caused by free fatty acids (FFA), chemokines, cytokines, or adipokines in NASH results in the release of endogenous molecules termed damage-associated molecular patterns (DAMPs).[7–9] Serum high mobility group box 1 protein (HMGB1), an evolutionarily conserved protein is a DAMP that serves to activate innate immunity and also act as a ligand for Toll-like receptors.[10] Although, HMGB1 was initially described as a late mediator of sepsis due to its releases from necrosis of cells, its role in sterile inflammation was subsequently recognized.[11] Therefore, it may also serve as an early mediator in the context of sterile inflammation that occurs in NASH.[7,12,13] Particularly, the switch of hepatic stellate cells toward a proinflammatory and profibrogenic phenotype through increased expression of chemokines such as monocyte chemoattractant protein-1 (MCP-1) and transforming growth factor beta 1(TGF-β) in NASH may be mediated through HMGB1.[14,15] There is some suggestion that HMGB1 may also regulate cellular process such as autophagy and apoptosis, two predominant mechanisms implicated in the pathophysiology of NASH.[16,17] Animal experiments reported HMGB1 release from hepatocytes in response to FFA infusion and subsequent treatment with neutralizing antibody to HMGB1 protected against FFA-induced tumor necrosis factor alpha and interleukin-6 production.[18]

Levels of circulating HMGB1 are elevated in patients with acute liver failure and acetaminophen-induced acute liver injury and indicative of the severity of liver injury.[19–21] The correlation between serum HMGB1 and histological severity of NAFLD as measured by NAFLD activity score (NAS) failed to show a consistent relationship in adult patients.[22] Unfortunately, the relationship between serum HMGB1 and fibrosis was not reported in this study.[22] The biomarker potential for serum HMGB1 levels in NAFLD was recently investigated in a large cohort of children with varying degrees of fibrosis in children with biopsy-proven NAFLD.[23] Plasma HMGB1 levels were higher in children with NAFLD than obese controls and correlated with the degree of fibrosis.[23] The levels were markedly elevated in those with clinically significant fibrosis (≥F2 stage of fibrosis) when compared to those without.[23] There was also a strong association with other biomarkers of fibrosis such as keratin 18 fragment levels and hyaluronic acid indicative of HMGB1-fibrosis relationship.[23] Although, there was no correlation with degree of hepatic inflammation, the correlation between levels of plasma HMGB1 levels and TGF-β and MCP-1 is suggestive of the critical role of HMGB1 in the pathogenesis of NAFLD.[23] Currently, there are no studies that examined the biomarker potential of HMGB1 in adult patients with NAFLD. Furthermore, it is not known if HMBG1 levels improve with treatment interventions and serve as a biomarker of treatment response. Testing the reliability and reproducibility of serum HMGB1 level as a biomarker for NAFLD severity is therefore very critical. In the current study, we investigated the relationship between NAFLD phenotype and treatment response on levels of serum HMGB1 in adults and children participating in PIVENS and TONIC clinical trials.

## Materials and methods

Biosamples archived (stored -70°C) from PIVENS and TONIC clinical trials were obtained through an ancillary study proposal submitted to the NASH Clinical Research Network (NASH CRN). The current study was reviewed by Indiana University Institutional Review Board (IRB). It was determined that IU IRB Review was not required (1604580147) since the authors did not have access to identifying information of the sample donors either as the physicians of the donors or upon access to the samples. Study design, demographic details, and clinical trial endpoints in these two trials have been previously published.[24,25] Briefly, in the PIVENS trial, the efficacy of daily pioglitazone (30mg) or vitamin E (800 IU), vs. placebo was assessed in 247 non-diabetic, adult patients with histologically defined NASH with a primary endpoint of overall improvement in liver histology at week 96 compared to baseline.[26] In the TONIC trial, the efficacy of vitamin E (800 IU/day), metformin (1000 mg/day) or placebo in 173 children with biopsy-proven NAFLD with sustained reduction in serum alanine aminotransferase (ALT) levels as the primary endpoint.[24] The overall improvement in liver histology at week 96 as compared to baseline was also assessed.[24] The clinical trials were approved by the review board at each participating center, and all subjects gave written informed consent.[27,28] Informed consent provided by the participants allowed for ancillary studies to be conducted on archived biological samples at a later point. We have included selected baseline characteristics for the PIVENS and TONIC patients with a serum HMGB1 measured at either baseline or 96 weeks who comprised the study population (S1 Table). Baseline and 96-week liver histology from both the studies was centrally scored by NASH CRN Pathology Committee (10 hepatopathologists blinded to clinical and treatment data) according to the previously published NASH CRN histological scoring system.[29] Briefly, the following histologic data were analyzed and diagnosis rendered by the Pathology Committee (i.e. “not steatohepatitis”, “borderline, zone 3 pattern”, “definite steatohepatitis”); the aggregate NAFLD activity score (NAS); the score of each component of the NAS (steatosis (0–3), lobular inflammation (0–3), ballooning (0–2)), and fibrosis scores (0,1a,1b,1c,2,3).[30] The serum HMGB1 concentration was measured by ELISA following the manufacturer's protocol (IBL International, Hamburg, Germany). The absorbance was determined at 450nm with the Vmax Kinetic Microplate reader by Molecular Devices M2 (Sunnyvale, CA). The standard curve and HMGB1 values were determined using the Soft-max Pro software (version 6.2) accompanying the microplate reader.

### Statistical analysis

In separate analyses for adults with NASH (PIVENS trial) and children with NAFLD (TONIC trial), serum HMGB1 levels were compared with histologic features, and *P* values were derived from linear regression of the rank of the serum HMGB1 values on various histological features. Multiple linear regression models adjusting for the baseline HMGB1 value were used to assess differences between mean changes of HMGB1 levels at 96 weeks from baseline between different comparator groups. *P* values for the differences in the serum HMGB1 time trends by treatment group or histological improvement were derived from separate multiple linear regression models for serum HMGB1 change in relation to independent variables as follows: baseline serum HMGB1 level, indicator variables for the classification group of interest, spline-type indicator variables for each time period, and interaction terms to allow the between group differences to vary with time; these regression models also included the use of generalized estimating equations (GEE) with robust variance estimation to account for within-patient correlations in repeated HMGB1 measures. All *P* values were 2-sided and nominal, and a *P* value <0.05 was considered statistically significant. The Stata 12 software (version 12.1, StatCorp, Cary, NC) and SAS version 9.3 (SAS Institute Inc., College Station, TX) were used for the statistical analyses.

## Results

### Study population

Out of 243 adult patients with NASH, who participated in the PIVENS trial, 125 serum samples from baseline, and 207 samples from week 96 were available for the current study. The median (IQR) duration between liver biopsy and sample used for measuring serum HMGB1 was 49 (23, 95) days at baseline, whereas it was only 2 (1, 9) days at the 96-week visit. Out of 173 children and adolescents who participated in the TONIC trial, 164 serum samples from baseline, and 109 samples from week 96 were available for the current study. The median (IQR) duration between liver biopsy and sample for serum HMGB1 measurement was 29 (14, 58) days at baseline and was only 2 (1, 2) days at the 96-week visit. The Spearman’s rank coefficient (rho) showed no correlation between adult or pediatric HOMA-IR (insulin resistance) and HMGB1, or BMI for adults, or leptin for children.

### Cross-sectional relationship between serum HMGB1 levels and histological severity of NAFLD

Due to the proximity of the liver biopsy and serum HMGB1 measurements, our cross-sectional analyses were undertaken on HMGB1 measurements and liver histology from the 96-week visit. There were no significant relationships between serum HMGB1 levels and a histological diagnosis of steatohepatitis, stage of fibrosis, grade of steatosis or severity of lobular inflammation, hepatocyte ballooning, and portal inflammation among the 207 adults who underwent liver biopsy at the conclusion of the PIVENS trial (Table 1). Similarly, serum HMGB1 levels were not associated with a histological diagnosis of steatohepatitis, fibrosis stage, steatosis grade or severity of lobular inflammation, hepatocyte ballooning and portal inflammation among 109 children who underwent liver biopsy at the conclusion of the TONIC trial (Table 1).

**Table 1:** Cross-sectional relationship between serum HMGB1 and various histological features in adults and children with NAFLD.

Histological feature	PIVENS (N = 207)		P*	TONIC (N = 109)		P*
	No	Mean ± SD (ng/mL)		No.	Mean ± SD (ng/mL)	
Fibrosis stage:			0.48			0.31
None	69	1.2 ± 1.8		38	1.4 ± 2.1	
Mild	65	2.0 ± 2.8		35	2.0 ± 3.6	
Moderate	37	1.5 ± 1.9		17	0.8 ± 1.7	
Bridging	27	1.9 ± 2.5		17	0.9 ± 1.3	
Cirrhosis	9	2.1 ± 2.6		0	--	
Steatosis grade:			0.97			0.28
≤ 33%	12	1.7 ± 2.4		50	1.1 ± 1.7	
34–66%	52	1.6 ± 2.1		26	1.5 ± 2.3	
> 66%	26	1.4 ± 2.1		31	1.9 ± 3.7	
Lobular inflammation:			0.94			0.29
< 2 foci	16	1.6 ± 2.3		70	1.5 ± 2.9	
≥ 2 foci	45	1.8 ± 2.4		37	1.3 ± 1.8	
Hepatocellular ballooning:			0.94			0.40
None	93	1.7 ± 2.6		61	1.6 ± 2.9	
Few	47	1.3 ± 2.0		29	1.8 ± 2.4	
Many	65	1.7 ± 2.1		17	0.6 ± 1.0	
Portal inflammation:			0.49			0.74
None	28	1.6 ± 2.3		17	1.0 ± 1.7	
Few	14	1.6 ± 2.3		76	1.5 ± 2.8	
Many	37	1.9 ± 2.3		14	1.4 ± 2.2	
Steatohepatitis diagnosis:			0.87			0.87
None	73	1.7 ± 2.6		46	1.1 ± 1.7	
Borderline suspicious zone 3	45	1.5 ± 2.3		20	1.9 ± 2.4	
Borderline, suspicious zone 1	0	--		10	3.5 ± 5.9	
Definite	87	1.7 ± 2.1		31	1.0 ± 1.6	

* HMGB1 levels assayed from the PIVENS and TONIC participants’ serum collected at 96 weeks.

P values (2-sided) for the association of histological feature and HMGB1 were derived from linear regression of the rank of the HMGB1 value on the histological feature.

### Relationship between different treatments and changes in serum HMGB1 levels

**Fig 1:**
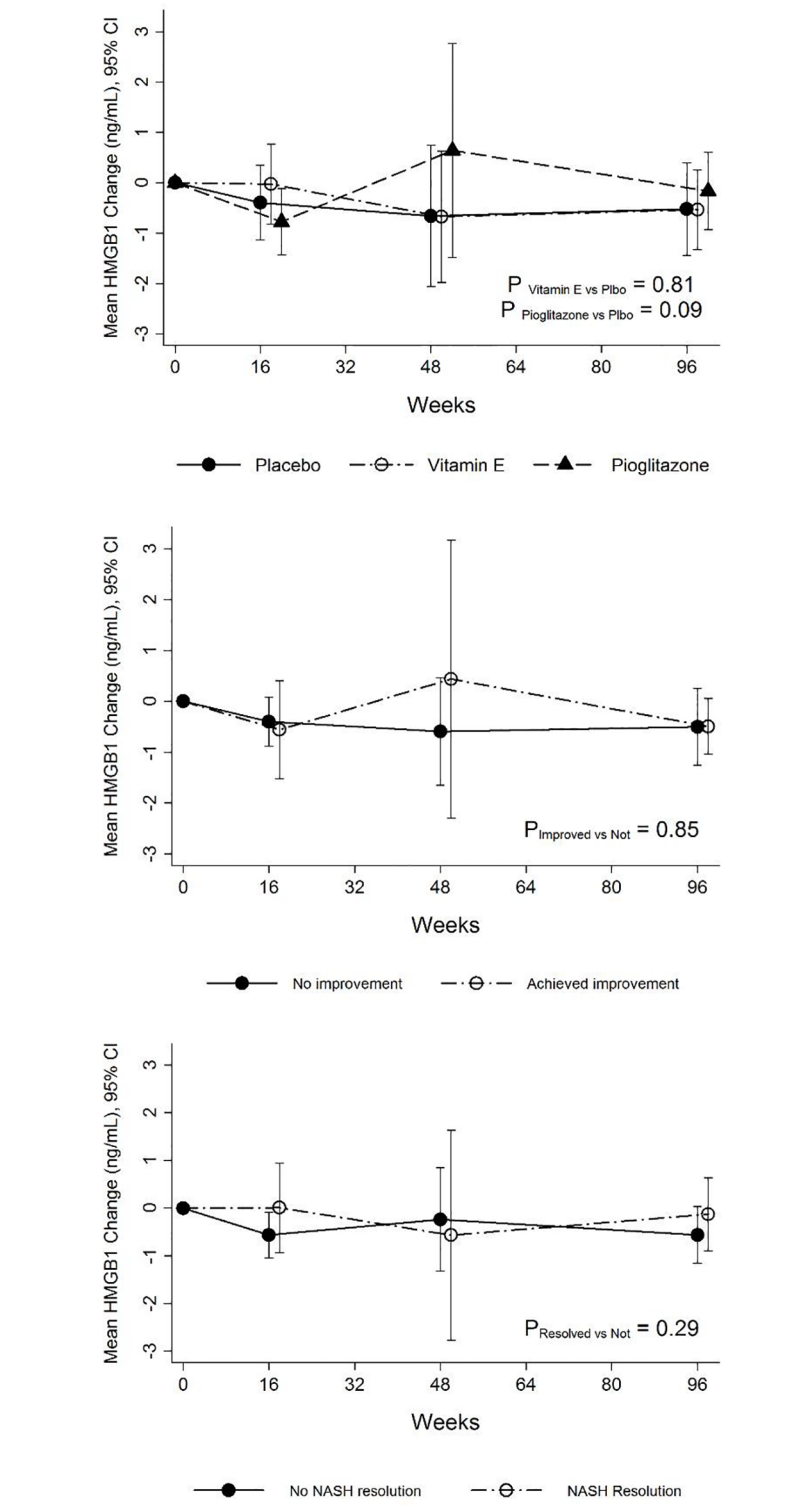
Serum HMGB1 levels during the PIVENS trial in the three treatment groups. At baseline, serum HMGB1 levels among the three treatment groups were similar. **Top panel:** Serum HMGB1 levels did not change significantly during treatment either with placebo, vitamin E therapy (*P* = 0.81) or pioglitazone (*P* = 0.09). **Middle panel:** Serum HMGB1 levels did not differ among individuals with or without histological improvement irrespective of treatment assignment during study duration (*P* = 0.85). **Bottom panel:** Similarly, serum HMGB1 levels did not differ among individuals with or without resolution of NASH irrespective of treatment assignment during study duration (*P* = 0.29).

In the subset of 105 patients in the PIVENS trial and 109 patients in the TONIC trial with paired samples from baseline and 96 weeks, the effect of different treatments on serum HMGB1 levels was examined. In the PIVENS trial, serum HMGB1 levels did not change significantly during treatment with placebo, vitamin E therapy (*P* = 0.81) or pioglitazone (*P* = 0.09) (Fig 1, top panel). Serum HMGB1 levels did not differ between (a) histological improvement vs. no improvement (*P* = 0.85, Fig 1, middle panel) and (b) resolution of NASH vs. no resolution (*P* = 0.29, Fig 1, bottom panel). At 96 weeks, serum HMGB1 levels did not differ among individuals in three different treatment arms (Vitamin E vs. Placebo: *P* = 0.83 and Pioglitazone vs. Placebo: P = 0.84) (Table 2).

**Table 2:** Change in serum HMGB1 in paired samples (baseline and 96 weeks) in PIVENS and TONIC participants by Treatment Group.

Clinical trial	Mean (± SD) change in HMGB1 from baseline to 96 weeks (ng/mL)			P*	
	Treatment group				
**PIVENS**	**Placebo (n = 42)**	**Pioglitazone (n = 42)**	**Vitamin E (n = 41)**	**Vitamin E vs. Placebo**	**Pioglitazone vs. Placebo**
	-0.5 ± 2.6	-0.2 ± 2.1	-0.5 ± 2.2	0.83	0.84
**TONIC**	**Placebo (n = 32)**	**Metformin (n = 40)**	**Vitamin E (n = 37)**	**Vitamin E vs. Placebo**	**Metformin vs. Placebo**
	-0.1 ± 1.9	-0.2 ± 2.1	-0.7 ± 3.4	0.29	0.91

* For the mean change in scores, P values were calculated with multiple linear regression models with two indicator variables for the effect of treatment versus placebo, adjusting for the baseline value of the outcome.

**Fig 2:**
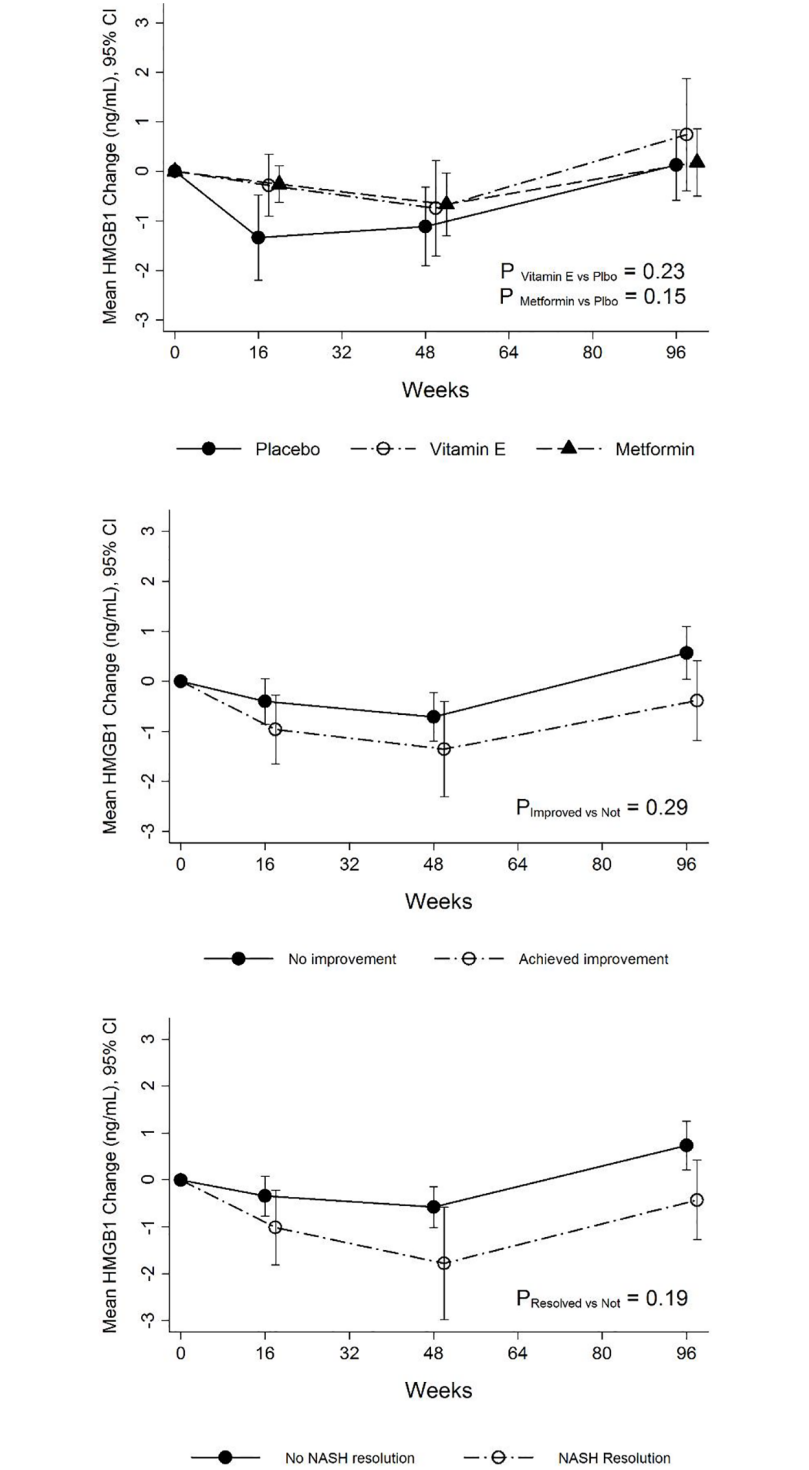
Serum HMGB1 levels during the TONIC trial in the three treatment groups. At baseline, serum HMGB1 levels among the three treatment groups were similar. **Top panel:** serum HMGB1 levels did not change significantly during treatment either with placebo, metformin (*P* = 0.15) or vitamin E (*P* = 0.23) therapy. **Middle panel:** Serum HMGB1 levels did not differ among individuals with or without treatment response (per protocol) during study duration (*P* = 0.29), irrespective of treatment assignment. **Bottom panel:** Similarly, serum HMGB1 levels did not differ among individuals during study duration with or without resolution of NASH (*P* = 0.19) irrespective of treatment assignment.

In the TONIC trial, serum HMGB1 levels did not change significantly during treatment with placebo, metformin (*P* = 0.15) or vitamin E (*P* = 0.23) therapy (Fig 2, top panel). Serum HMGB1 levels decreased with both metformin and vitamin E at week 96, but once again the decrease was not significantly different when compared to the placebo arm (Table 2). Serum HMGB1 levels did not differ among individuals with or without treatment response (per protocol) during the clinical trial (*P* = 0.29), irrespective of treatment assignment (Fig 2, middle panel). Similarly, serum HMGB1 levels did not differ among individuals with or without resolution of NASH (*P* = 0.19) irrespective of treatment assignment (Fig 2, bottom panel).

### Relationship between changes in serum HMGB1 levels and histological response irrespective of treatment assignment

In the PIVENS trial, serum HMGB1 levels were not significantly different between patients with and without histologic response (*P* = 0.90) or with or without resolution of NASH (*P* = 0.30) (Fig 1 middle panel and bottom panel). The mean change in the serum HMGB1 levels at week 96 irrespective of treament assignment was not statistically significantly different (-0.5 ± 1.5 vs. -0.5 ± 2.5 ng/mL, *P* = 0.22) (S2 Table) between the patients who did and did not achieve histological improvement and with or without resolution of NASH (No NASH resolution vs. NASH resolution: -0.6 ± 2.5 vs. -0.1 ± 2.2 ng/mL, *P* = 0.57) (S3 Table). In the TONIC trial, serum HMGB1 levels between patients during the trial were not significantly different for overall histologic response (*P* = 0.30) (Fig 2 and S4 Table) or resolution of NASH (*P* = 0.20) (Fig 2 and S5 Table). The mean change in serum HMGB1 levels after 96 weeks of therapy was -0.4 ± 2.4 ng/mL in those with histologic improvement compared to 0.6 ± 2.1 ng/mL in those without (*P =* 0.32) (S4 Table). The mean change in serum HMGB1 levels after 96 weeks of therapy was -0.4 ± 2.6 ng/mL in those with NASH resolution compared to 0.7 ± 1.8 ng/mL in those without (*P =* 0.15) (S5 Table).

### Relationship between serum HMGB1 levels and serum ALT levels

In the PIVENS trial, there was no relationship between serum ALT and HMGB1 levels [change in serum ALT for every 10 ng/mL decrease in HMGB1 was -0.1 (-0.4, 0.1), *P* = 0.33]. In the TONIC trial as well, there was no relationship between serum ALT and HMGB1 levels [change in serum ALT for every 10 ng/mL decrease in HMGB1 was -0.3 (-0.8, 0.2), *P* = 0.24].

## Discussion

Studies that examined the relationship between serum HMGB1 and fibrotic disorders such as idiopathic pulmonary fibrosis and systemic sclerosis have yielded contrasting results.[31,32] Studies that examined the relationship between serum and tissue levels of HMGB1 have also failed to show any consistent relationship.[31,32]

Sterile inflammation and subsequent release of DAMPs from hepatocyte injury are key processes in the pathophysiology of NASH. The significant association between increased levels of plasma HMGB1 and a higher degree of liver fibrosis in the recent pediatric study highlighted its biomarker potential for non-invasive diagnosis of NASH.[23] However, in the current study, we failed to show any significant relationship between serum HMGB1 levels and degree of liver fibrosis or histologic severity in either children or adults with biopsy-proven NAFLD. Moreover, changes in serum HMGB1 levels did not differ significantly with vitamin E, metformin or pioglitazone therapy when compared to placebo. Irrespective of treatment assignment, changes in serum HMGB1 levels did not differ in those with or without a treatment response or resolution of NASH. The biomarker potential of serum HMGB1 for the diagnosis of NAFLD in children could not be evaluated in the current study as the study design of TONIC precluded enrollment of children without NAFLD.

Lack of relationship between serum HMGB1 and degree of fibrosis in the current study is in contrast to previously published study despite similar sample size, range of values, and assay used for measurement serum HMGB1 levels. Although perplexing, the discrepancy may be related to differences in the NAFLD phenotype; the current study included 32% advanced fibrosis vs. 18% in the Italian study.[23] The lack of association in the with both adults and children although disappointing, highlights the challenges in understanding the relationship between serum levels of DAMPs and understand their role in NASH pathogenesis.[33] Moreover, circulating HMGB1 and liver HMGB1 could represent two different pools and further studies that simultaneously quantify the hepatic and peripheral HMGB1to examine the relationship between DAMPs and NASH pathogenesis are awaited.

Single blood biomarker to predict the presence or severity of NAFLD is inherently challenging due to its complex pathophysiology. Caution should be exercised when using a single biomarker to predict disease severity. To better comprehend the findings from the present study, factors that could alter HMGB1 function and levels would merit further discussion. The cellular source for HMGB1 is the nucleus and its acetylation in the cytoplasm stops its reentry into the nucleus with subsequent migration into cytoplasmic secretory vesicles.[34] HMGB1 is also sensitive to the redox state and is rapidly inactivated in the normal oxidative extracellular environment.[35] In general, it is well accepted that innate immune cells secrete the acetylated form (active secretion), and necrotic dying cells release non acetylated form.[36] Future studies should consider measuring the acetylated form and examine if these levels are down-regulated with NASH resolution. Also, studies have also shown that short-term fasting causes a reduction in circulating HMGB1 due to cytoplasmic HMGB1 translocation and induction of autophagy.[17] In the current study, the mean serum levels of HMGB1 appear to be lower than the previously reported values in literature despite similar methodology to our previously published work. [23,37]

In summary, serum HMGB1 levels did not correlate with NAFLD severity or improvements in liver histology or serum ALT. We speculate that one or a combination of factors could perhaps explain the lack the relationship between serum HMGB1 and NAFLD severity. Future studies could perhaps either measure acetylated HGMB1, HMGB1 isoforms or measure in a non-fasting state with a standardized meal.

## Supporting information

S1 Table Baseline characteristics of adults and children in the HMGB1 study. (DOCX)

S2 Table Baseline level and change in HMGB1 at 16, 48 and 96 weeks of follow-up in PIVENS participants by overall histological improvement. (DOCX)

S3 Table Baseline level and change in HMGB1 at 16, 48 and 96 weeks of follow-up in PIVENS participants by resolution in NASH. (DOCX)

S4 Table Baseline level and change in HMGB1 at 16, 48 and 96 weeks of follow-up in TONIC participants by overall histological improvement. (DOCX)

S5 Table Baseline level and change in HMGB1 at 16, 48 and 96 weeks of follow-up in TONIC participants by resolution in NASH. (DOCX)

## References

[1] Browning JD, Szczepaniak LS, Dobbins R, Nuremberg P, Horton JD, et al (2004) Prevalence of hepatic steatosis in an urban population in the United States: impact of ethnicity. Hepatology 40: 1387–1395. doi: 10.1002/hep.20466 15565570 10.1002/hep.20466

[2] Loomba R, Sirlin CB, Schwimmer JB, Lavine JE (2009) Advances in pediatric nonalcoholic fatty liver disease. Hepatology 50: 1282–1293. doi: 10.1002/hep.23119 19637286 10.1002/hep.23119 PMC2757471

[3] Mencin AA, Lavine JE (2011) Advances in pediatric nonalcoholic fatty liver disease. Pediatr Clin North Am 58: 1375–1392, x doi: 10.1016/j.pcl.2011.09.005 22093857 10.1016/j.pcl.2011.09.005

[4] Molleston JP, Schwimmer JB, Yates KP, Murray KF, Cummings OW, et al (2014) Histological abnormalities in children with nonalcoholic fatty liver disease and normal or mildly elevated alanine aminotransferase levels. J Pediatr 164: 707–713 e703 doi: 10.1016/j.jpeds.2013.10.071 24360992 10.1016/j.jpeds.2013.10.071 PMC3962701

[5] Neuschwander-Tetri BA, Clark JM, Bass NM, Van Natta ML, Unalp-Arida A, et al (2010) Clinical, laboratory and histological associations in adults with nonalcoholic fatty liver disease. Hepatology 52: 913–924. doi: 10.1002/hep.23784 20648476 10.1002/hep.23784 PMC3070295

[6] Sanyal AJ, Brunt EM, Kleiner DE, Kowdley KV, Chalasani N, et al (2011) Endpoints and clinical trial design for nonalcoholic steatohepatitis. Hepatology 54: 344–353. doi: 10.1002/hep.24376 21520200 10.1002/hep.24376 PMC4014460

[7] Ganz M, Szabo G (2013) Immune and inflammatory pathways in NASH. Hepatol Int 7: 771–781. doi: 10.1007/s12072-013-9468-6 24587847 10.1007/s12072-013-9468-6 PMC3918407

[8] Frazier TH, DiBaise JK, McClain CJ (2011) Gut microbiota, intestinal permeability, obesity-induced inflammation, and liver injury. JPEN J Parenter Enteral Nutr 35: 14S–20S. doi: 10.1177/0148607111413772 21807932 10.1177/0148607111413772

[9] Chait A, Kim F (2010) Saturated fatty acids and inflammation: who pays the toll? Arterioscler Thromb Vasc Biol 30: 692–693. doi: 10.1161/ATVBAHA.110.203984 20237329 10.1161/ATVBAHA.110.203984

[10] Takeda K, Kaisho T, Akira S (2003) Toll-like receptors. Annu Rev Immunol 21: 335–376. doi: 10.1146/annurev.immunol.21.120601.141126 12524386 10.1146/annurev.immunol.21.120601.141126

[11] Scaffidi P, Misteli T, Bianchi ME (2002) Release of chromatin protein HMGB1 by necrotic cells triggers inflammation. Nature 418: 191–195. doi: 10.1038/nature00858 12110890 10.1038/nature00858

[12] Alisi A, Carsetti R, Nobili V (2011) Pathogen- or damage-associated molecular patterns during nonalcoholic fatty liver disease development. Hepatology 54: 1500–1502. doi: 10.1002/hep.24611 22045668 10.1002/hep.24611

[13] Gauley J, Pisetsky DS (2009) The translocation of HMGB1 during cell activation and cell death. Autoimmunity 42: 299–301. 19811282 10.1080/08916930902831522

[14] Zhang Z, Lin C, Peng L, Ouyang Y, Cao Y, et al (2012) High mobility group box 1 activates Toll like receptor 4 signaling in hepatic stellate cells. Life Sci 91: 207–212. doi: 10.1016/j.lfs.2012.07.009 22841886 10.1016/j.lfs.2012.07.009

[15] Lenga Y, Koh A, Perera AS, McCulloch CA, Sodek J, et al (2008) Osteopontin expression is required for myofibroblast differentiation. Circ Res 102: 319–327. doi: 10.1161/CIRCRESAHA.107.160408 18079410 10.1161/CIRCRESAHA.107.160408

[16] Saitoh T, Fujita N, Jang MH, Uematsu S, Yang BG, et al (2008) Loss of the autophagy protein Atg16L1 enhances endotoxin-induced IL-1beta production. Nature 456: 264–268. doi: 10.1038/nature07383 18849965 10.1038/nature07383

[17] Rickenbacher A, Jang JH, Limani P, Ungethum U, Lehmann K, et al (2014) Fasting protects liver from ischemic injury through Sirt1-mediated downregulation of circulating HMGB1 in mice. J Hepatol 61: 301–308. doi: 10.1016/j.jhep.2014.04.010 24751831 10.1016/j.jhep.2014.04.010

[18] Zhang W, Wang LW, Wang LK, Li X, Zhang H, et al (2013) Betaine protects against high-fat-diet-induced liver injury by inhibition of high-mobility group box 1 and Toll-like receptor 4 expression in rats. Dig Dis Sci 58: 3198–3206. doi: 10.1007/s10620-013-2775-x 23861108 10.1007/s10620-013-2775-x

[19] Oshima G, Shinoda M, Tanabe M, Ebinuma H, Nishiyama R, et al (2012) Increased plasma levels of high mobility group box 1 in patients with acute liver failure. Eur Surg Res 48: 154–162. doi: 10.1159/000338363 22585050 10.1159/000338363

[20] Antoine DJ, Dear JW, Lewis PS, Platt V, Coyle J, et al (2013) Mechanistic biomarkers provide early and sensitive detection of acetaminophen-induced acute liver injury at first presentation to hospital. Hepatology 58: 777–787. doi: 10.1002/hep.26294 23390034 10.1002/hep.26294 PMC3842113

[21] Oshima G, Shinoda M, Tanabe M, Ebinuma H, Nishiyama R, et al (2012) Increased plasma levels of high mobility group box 1 in patients with acute liver failure. Eur Surg Res 48: 154–162. doi: 10.1159/000338363 22585050 10.1159/000338363

[22] Grove JI, Antoine DJ, Kaye P, Miller MH, Dillon JF, et al (2014) DO SERUM MARKERS OF CELL INJURY AND DEATH HAVE POTENTIAL TO BECOME MECHANISTIC MARKERS IN NON-ALCOHOLIC FATTY LIVER DISEASE (NAFLD)? Gut 63: A245–A246.

[23] Alisi A, Nobili V, Ceccarelli S, Panera N, De Stefanis C, et al (2014) Plasma high mobility group box 1 protein reflects fibrosis in pediatric nonalcoholic fatty liver disease. Expert Rev Mol Diagn 14: 763–771. doi: 10.1586/14737159.2014.928205 24927058 10.1586/14737159.2014.928205

[24] Lavine JE, Schwimmer JB, Van Natta ML, Molleston JP, Murray KF, et al (2011) Effect of vitamin E or metformin for treatment of nonalcoholic fatty liver disease in children and adolescents: the TONIC randomized controlled trial. JAMA 305: 1659–1668. doi: 10.1001/jama.2011.520 21521847 10.1001/jama.2011.520 PMC3110082

[25] Chalasani NP, Sanyal AJ, Kowdley KV, Robuck PR, Hoofnagle J, et al (2009) Pioglitazone versus vitamin E versus placebo for the treatment of non-diabetic patients with non-alcoholic steatohepatitis: PIVENS trial design. Contemp Clin Trials 30: 88–96. doi: 10.1016/j.cct.2008.09.003 18804555 10.1016/j.cct.2008.09.003 PMC2929909

[26] Sanyal AJ, Chalasani N, Kowdley KV, McCullough A, Diehl AM, et al (2010) Pioglitazone, vitamin E, or placebo for nonalcoholic steatohepatitis. N Engl J Med 362: 1675–1685. doi: 10.1056/NEJMoa0907929 20427778 10.1056/NEJMoa0907929 PMC2928471

[27] Sanyal AJ, Chalasani N, Kowdley KV, McCullough A, Diehl AM, et al (2010) Pioglitazone, vitamin E, or placebo for nonalcoholic steatohepatitis. N Engl J Med 362: 1675–1685. doi: 10.1056/NEJMoa0907929 20427778 10.1056/NEJMoa0907929 PMC2928471

[28] Lavine JE, Schwimmer JB, Van Natta ML, Molleston JP, Murray KF, et al (2011) Effect of vitamin E or metformin for treatment of nonalcoholic fatty liver disease in children and adolescents: the TONIC randomized controlled trial. JAMA 305: 1659–1668. doi: 10.1001/jama.2011.520 21521847 10.1001/jama.2011.520 PMC3110082

[29] Kleiner DE, Brunt EM, Van Natta M, Behling C, Contos MJ, et al (2005) Design and validation of a histological scoring system for nonalcoholic fatty liver disease. Hepatology 41: 1313–1321. doi: 10.1002/hep.20701 15915461 10.1002/hep.20701

[30] Kleiner DE, Brunt EM, Van Natta M, Behling C, Contos MJ, et al (2005) Design and validation of a histological scoring system for nonalcoholic fatty liver disease. Hepatology 41: 1313–1321. doi: 10.1002/hep.20701 15915461 10.1002/hep.20701

[31] Yoshizaki A, Komura K, Iwata Y, Ogawa F, Hara T, et al (2009) Clinical significance of serum HMGB-1 and sRAGE levels in systemic sclerosis: association with disease severity. J Clin Immunol 29: 180–189. doi: 10.1007/s10875-008-9252-x 18825489 10.1007/s10875-008-9252-x

[32] Hamada N, Maeyama T, Kawaguchi T, Yoshimi M, Fukumoto J, et al (2008) The role of high mobility group box1 in pulmonary fibrosis. Am J Respir Cell Mol Biol 39: 440–447. doi: 10.1165/rcmb.2007-0330OC 18441281 10.1165/rcmb.2007-0330OC

[33] Kubes P, Mehal WZ (2012) Sterile inflammation in the liver. Gastroenterology 143: 1158–1172. doi: 10.1053/j.gastro.2012.09.008 22982943 10.1053/j.gastro.2012.09.008

[34] Bonaldi T, Talamo F, Scaffidi P, Ferrera D, Porto A, et al (2003) Monocytic cells hyperacetylate chromatin protein HMGB1 to redirect it towards secretion. EMBO J 22: 5551–5560. doi: 10.1093/emboj/cdg516 14532127 10.1093/emboj/cdg516 PMC213771

[35] Venereau E, Casalgrandi M, Schiraldi M, Antoine DJ, Cattaneo A, et al (2012) Mutually exclusive redox forms of HMGB1 promote cell recruitment or proinflammatory cytokine release. J Exp Med 209: 1519–1528. doi: 10.1084/jem.20120189 22869893 10.1084/jem.20120189 PMC3428943

[36] Antoine DJ, Harris HE, Andersson U, Tracey KJ, Bianchi ME (2014) A systematic nomenclature for the redox states of high mobility group box (HMGB) proteins. Mol Med 20: 135–137. doi: 10.2119/molmed.2014.00022 24531895 10.2119/molmed.2014.00022 PMC3966994

[37] Masuoka HC, Vuppalanchi R, Deppe R, Bybee P, Comerford M, et al (2015) Individuals with Primary Sclerosing Cholangitis Have Elevated Levels of Biomarkers for Apoptosis but Not Necrosis. Dig Dis Sci 60: 3642–3646. doi: 10.1007/s10620-015-3805-7 26195313 10.1007/s10620-015-3805-7 PMC4637218

