# Supplementary material for: Serum high mobility group box 1 protein levels are not associated with either histological severity or treatment response in children and adults with nonalcoholic fatty liver disease
Source: PLoS One. 2017 Nov 2;12(11):e0185813. doi: 10.1371/journal.pone.0185813 (PMC5667763; doi:10.1371/journal.pone.0185813)
Supplement: S1 Table — (DOCX) [file pone.0185813.s001.docx]

**S1 Table. Baseline characteristics of adults and children in the HMGB1 study**

| **Characteristic** | **PIVENS***  **(N=207)** |  | **TONIC***  **(N=109)** |
| --- | --- | --- | --- |
| **Demographic** |  |  |  |
| Age (years) | 47.1 (12.1) |  | 13.1 (2.3) |
| Male | 84 (41%) |  | 89 (82%) |
| Race: |  |  |  |
| White | 173 (86%) |  | 79 (80%) |
| Black | 3 (2%) |  | 0 (0%) |
| Other | 24 (12%) |  | 20 (20%) |
| Hispanic ethnicity | 25 (12%) |  | 76 (70%) |
| **Anthropometric** |  |  |  |
| BMI (kg/m^2^) | 33.9 (6.4) |  |  |
| BMI Z-score |  |  | 2.4 (0.3) |
| Waist circumference (cm) | 107.3 (13.4) |  | 108.5 (14.1) |
| **HMGB1† (ng/mL)** | 1.56 (1.80) |  | 1.10 (1.93) |
| **Laboratory measures** |  |  |  |
| ALT (U/L) | 82.4 (48.1) |  | 122.5 (62.5) |
| AST (U/L) | 56.2 (29.3) |  | 72.6 (43.7) |
| GGT (U/L) | 57.8 (57.4) |  | 48.2 (30.7) |
| Glucose (mg/dL) | 93.6 (13.4) |  | 89.3 (9.1) |
| Insulin | 22.4 (18.7) |  | 46.4 (57.2) |
| HOMA-IR | 5.3 (4.6) |  | 10.4 (14.0) |
| Triglycerides (mg/dL) | 167.7 (94.6) |  | 144.5 (83.9) |
| Cholesterol, total (mg/dL) | 198.9 (39.4) |  | 173.4 (34.7) |
| Cholesterol, HDL (mg/dL) | 43.7 (11.6) |  | 36.7 (7.8) |
|  |  |  |  |

* Adults and children are included if there is a serum HMGB1 value measured.

†HMGB1 value measured at baseline for 98 PIVENS subjects and 105 TONIC.
