# Supplementary material for: Serum high mobility group box 1 protein levels are not associated with either histological severity or treatment response in children and adults with nonalcoholic fatty liver disease
Source: PLoS One. 2017 Nov 2;12(11):e0185813. doi: 10.1371/journal.pone.0185813 (PMC5667763; doi:10.1371/journal.pone.0185813)
Supplement: S2 Table — (DOCX) [file pone.0185813.s002.docx]

**S2 Table: Baseline level and change in HMGB1 at 16, 48 and 96 weeks of follow-up in PIVENS participants by overall histological improvement**

|  | **Overall Histological Improvement*** | | | | ***P***† |
| --- | --- | --- | --- | --- | --- |
|  | **No improvement** | | **Achieved improvement** | | **Improved vs Not improved** |
| **HMGB1 (ng/mL) at week:** | (n) |  | (n) |  |  |
| Baseline | 63 | 1.63 ± 1.73 | 36 | 1.44 ± 1.46 | 0.58 |
| 16 weeks | 45 | 1.32 ± 1.53 | 31 | 1.46 ± 2.17 | 0.75 |
| 48 weeks | 62 | 0.90 ± 1.23 | 49 | 1.29 ± 1.94 | 0.19 |
| 96 weeks | 92 | 1.69 ± 2.09 | 72 | 1.72 ± 2.29 | 0.93 |
| **Mean change from baseline:** |  |  |  |  |  |
| After 16 weeks of therapy | 29 | -0.40 ± 1.27 | 15 | -0.56 ± 1.74 | 0.71 |
| After 48 weeks of therapy | 20 | -0.59 ± 2.26 | 7 | 0.44 ± 2.95 | 0.66 |
| After 96 weeks of therapy | 45 | -0.51 ± 2.51 | 31 | -0.50 ± 1.50 | 0.22 |

*Overall Histologic Response required improvement by 1 or more points in the hepatocellular ballooning score; no increase in the fibrosis score; and either a decrease in the activity score for nonalcoholic fatty liver disease to a score of 3 points or less or a decrease in the activity score of at least 2 points, with at least a 1-point decrease in either the lobular inflammation or steatosis score.

† For the mean change in scores, P values were calculated with ANCOVA models with an indicator variable for overall histologic response, adjusting for the baseline value of the outcome
