# Supplementary material for: Serum high mobility group box 1 protein levels are not associated with either histological severity or treatment response in children and adults with nonalcoholic fatty liver disease
Source: PLoS One. 2017 Nov 2;12(11):e0185813. doi: 10.1371/journal.pone.0185813 (PMC5667763; doi:10.1371/journal.pone.0185813)
Supplement: S3 Table — (DOCX) [file pone.0185813.s003.docx]

**S3 Table: Baseline level and change in HMGB1 at 16, 48 and 96 weeks of follow-up in PIVENS participants by resolution in NASH**

|  | **Resolution in NASH** | | | | ***P**** |
| --- | --- | --- | --- | --- | --- |
|  | **No NASH resolution** | | **NASH resolution** | | **Resolved vs**  **Not Resolved** |
| **HMGB1 (ng/mL) at week:** | (n) |  | (n) |  |  |
| Baseline | 74 | 1.62 ± 1.71 | 37 | 1.42 ± 1.90 | 0.57 |
| 16 weeks | 55 | 1.13 ± 1.41 | 24 | 1.74 ± 2.40 | 0.16 |
| 48 weeks | 85 | 0.96 ± 1.36 | 44 | 1.08 ± 1.88 | 0.67 |
| 96 weeks | 132 | 1.60 ± 2.14 | 73 | 1.72 ± 2.56 | 0.72 |
| **Mean change from baseline:** |  |  |  |  |  |
| After 16 weeks of therapy | 33 | -0.57 ± 1.34 | 13 | -0.01 ± 1.55 | 0.29 |
| After 48 weeks of therapy | 23 | -0.24 ± 2.50 | 5 | -0.57 ± 1.77 | 0.12 |
| After 96 weeks of therapy | 64 | -0.56 ± 2.51 | 34 | -0.13 ± 2.20 | 0.57 |

*For the mean change in scores, P values were calculated with ANCOVA models with an indicator variable for resolution of NASH, adjusting for the baseline value of the outcome.
