# Supplementary material for: Serum high mobility group box 1 protein levels are not associated with either histological severity or treatment response in children and adults with nonalcoholic fatty liver disease
Source: PLoS One. 2017 Nov 2;12(11):e0185813. doi: 10.1371/journal.pone.0185813 (PMC5667763; doi:10.1371/journal.pone.0185813)
Supplement: S4 Table — (DOCX) [file pone.0185813.s004.docx]

**S4 Table: Baseline level and change in HMGB1 at 16, 48 and 96 weeks of follow-up in TONIC participants by overall histological improvement**

|  | **Overall Histological Improvement*** | | | | ***P***† |
| --- | --- | --- | --- | --- | --- |
|  | **No improvement** | | **Achieved improvement** | | **Improved vs Not improved** |
| **HMGB1 (ng/mL) at week:** | (n) |  | (n) |  |  |
| Baseline | 88 | 1.19 ± 1.78 | 52 | 1.49 ± 2.18 | 0.39 |
| 24 weeks | 74 | 0.68 ± 1.30 | 45 | 0.45 ± 0.70 | 0.28 |
| 48 weeks | 69 | 0.67 ± 1.10 | 37 | 0.42 ± 1.26 | 0.30 |
| 96 weeks | 68 | 1.31 ± 1.92 | 38 | 1.18 ± 1.75 | 0.74 |
| **Mean change from baseline:** |  |  |  |  |  |
| After 24 weeks of therapy | 73 | -0.40 ± 1.96 | 44 | -0.96 ± 2.26 | 0.28 |
| After 48 weeks of therapy | 69 | -0.71 ± 2.02 | 35 | -1.35 ± 2.78 | 0.15 |
| After 96 weeks of therapy | 66 | 0.57 ± 2.13 | 36 | -0.38 ± 2.37 | 0.32 |

* Overall Histologic Response required improvement no increase in the fibrosis score, and a decrease in the activity score for nonalcoholic fatty liver disease of at least 2 points.

†For the mean change in scores, P values were calculated with ANCOVA models with an indicator variable for overall histologic response, adjusting for the baseline value of the outcome.
