# Supplementary material for: Serum high mobility group box 1 protein levels are not associated with either histological severity or treatment response in children and adults with nonalcoholic fatty liver disease
Source: PLoS One. 2017 Nov 2;12(11):e0185813. doi: 10.1371/journal.pone.0185813 (PMC5667763; doi:10.1371/journal.pone.0185813)
Supplement: S5 Table — (DOCX) [file pone.0185813.s005.docx]

**S5 Table: Baseline level and change in HMGB1 at 16, 48 and 96 weeks of follow-up in TONIC participants by resolution in NASH**

|  | **Resolution in NASH** | | | | ***P**** |
| --- | --- | --- | --- | --- | --- |
|  | **No NASH resolution** | | **NASH resolution** | | **Resolved vs**  **Not Resolved** |
| **HMGB1 (ng/mL) at week:** | (n) |  | (n) |  |  |
| Baseline | 64 | 1.03 ± 1.47 | 52 | 1.60 ± 2.51 | 0.13 |
| 24 weeks | 55 | 0.56 ± 0.92 | 42 | 0.42 ± 0.63 | 0.41 |
| 48 weeks | 55 | 0.64 ± 1.08 | 31 | 0.28 ± 1.29 | 0.18 |
| 96 weeks | 50 | 1.36 ± 1.80 | 39 | 1.11 ± 1.74 | 0.53 |
| **Mean change from baseline:** |  |  |  |  |  |
| After 24 weeks of therapy | 53 | -0.35 ± 1.53 | 42 | -1.02 ± 2.56 | 0.43 |
| After 48 weeks of therapy | 54 | -0.58 ± 1.61 | 31 | -1.78 ± 3.27 | 0.20 |
| After 96 weeks of therapy | 47 | 0.73 ± 1.77 | 39 | -0.43 ± 2.61 | 0.15 |

* For the mean change in scores, P values were calculated with ANCOVA models with an indicator variable for resolution of NASH, adjusting for the baseline value of the outcome.
